# Microbial enzymes will offer limited solutions to the global plastic pollution crisis

**DOI:** 10.1111/1751-7915.14135

**Published:** 2022-09-13

**Authors:** Jennifer Chow, Pablo Perez‐Garcia, Robert Dierkes, Wolfgang R. Streit

**Affiliations:** ^1^ Department of Microbiology and Biotechnology University of Hamburg Hamburg Germany

## Abstract

Global economies depend on the use of fossil‐fuel‐based polymers with 360–400 million metric tons of synthetic polymers being produced per year. Unfortunately, an estimated 60% of the global production is disposed into the environment. Within this framework, microbiologists have tried to identify plastic‐active enzymes over the past decade. Until now, this research has largely failed to deliver functional biocatalysts acting on the commodity polymers such as polyethylene (PE), polypropylene (PP), polyvinylchloride (PVC), ether‐based polyurethane (PUR), polyamide (PA), polystyrene (PS) and synthetic rubber (SR). However, few enzymes are known to act on low‐density and low‐crystalline (amorphous) polyethylene terephthalate (PET) and ester‐based PUR. These above‐mentioned polymers represent >95% of all synthetic plastics produced. Therefore, the main challenge microbiologists are currently facing is in finding polymer‐active enzymes targeting the majority of fossil‐fuel‐based plastics. However, identifying plastic‐active enzymes either to implement them in biotechnological processes or to understand their potential role in nature is an emerging research field. The application of these enzymes is still in its infancy. Here, we summarize the current knowledge on microbial plastic‐active enzymes, their global distribution and potential impact on plastic degradation in industrial processes and nature. We further outline major challenges in finding novel plastic‐active enzymes, optimizing known ones by synthetic approaches and problems arising through falsely annotated and unfiltered use of database entries. Finally, we highlight potential biotechnological applications and possible re‐ and upcycling concepts using microorganisms.

## Graphical abstract

Plastics pollute the environment at all levels. Until now, microbiologist largely failed delivering functional biocatalysts acting on most of the the commodity polymers Therefore, identifying plastic‐active enzymes either to implement them in biotechnological processes or to understand their potential role in nature is an emerging research field. Here summarize knowledge on available enzymes and technologies to used for mining novel enzymes.

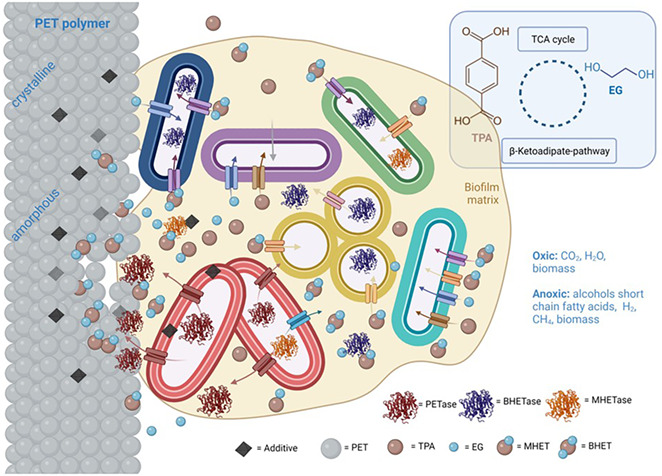

## INTRODUCTION AND BACKGROUND

**FIGURE 1:**
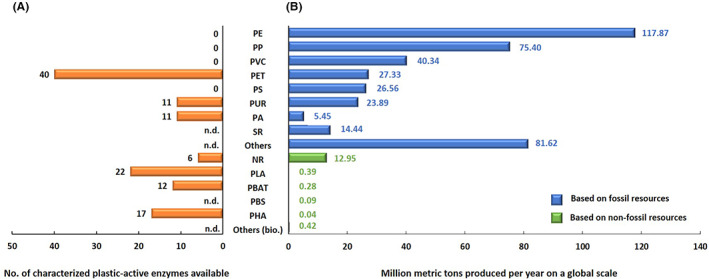
Main types of plastic polymers used in global bioeconomy. (A) Biochemically characterized enzymes available for the different commodity polymers. (B) Global annual production rates. Data are derived from european‐bioplastics.org; statiscia.com; iea.org; fao.org and pazy.eu and were downloaded from the various databases in February and March 2022.

At the end of the 1930s, the first synthetic and fossil‐fuel‐based polymers were developed and quickly found a wide range of applications in many areas of our daily lives, industries and society. Today, plastic materials have become indispensable in a wide range of applications due to their high stability, durability and a multitude of further positive properties. Notably, many plastic materials are often conceived for one‐time use only. As many as 360–400 million metric tons of synthetic polymers are produced worldwide every year (Plasticseurope, 2018). The vast majority of these (>95%) are based on fossil resources and they are often referred to as low‐cost, commodity thermoplastics. Figure 1 summarizes the most frequently used polymers and their global production rates. The main types of fossil fuel‐based polymers produced at the largest scales are polyethylene (PE), polypropylene (PP), polyvinylchloride (PVC), polyurethane (PUR), polyamide (nylon; PA), polyethylene terephthalate (PET), polystyrene (PS) and synthetic rubber (SR) (Figure 1, Table 1, Plasticsinsight, 2016, Plasticseurope, 2018, Statista.com, 2022).

**TABLE 1:** Known and verified hydrolases acting on the fossil‐fuel‐based polymers polyethylene terephthalate (PET) and ether‐based polyurethane (PUR). The table includes only wildtype enzymes, for which activities have been verified in biochemical tests. Predicted enzymes were excluded.

Microbial host/enzyme/gene	Reference
*(A) PET‐active enzymes*	
Proteobacteria	
*Ideonella sakaiensis* 201‐F6, *Is*PETase, *ISF6_4831*	Yoshida et al. (2016)
*Oleispira antarctica* RB‐8, PET5, LipA	Danso et al. (2018)
*Vibrio gazogenes*, PET6, *BSQ33_03270*	Danso et al. (2018)
*Polyangium brachysporum*, PET12, *AAW51_2473*	Danso et al. (2018)
*Pseudomonas pseudoalcaligenes* DSM 50188, PpCutA	Haernvall et al. (2017a), Inglis et al. (2011)
*Pseudomonas pelagia* DSM 25163, PpelaLip	Haernvall et al. (2017a)
*Pseudomonas aestusnigri* VGXO14, PE‐H, *B7O88_11480*	Bollinger et al. (2020)
*Pseudomonas mendocina* ATCC 53552, PmC	Ronkvist et al. (2009)
*Moraxella* sp. TA144, lip1, Mors1	Danso et al. (2018)
*Pseudomonas pseudoalcaligenes*, PpEst (tesA)	Haernvall et al. (2017b), Wallace et al. (2017)
Actinobacteria	
LCC, leaf compost metagenome	Sulaiman et al. (2012)
BhrPETase from HR29 bacterium, >96% identical to LCC	Xi et al. (2021)
*Thermobifida (Thermomonospora) fusca* DSM43793, BTA‐1, TfH	Kleeberg et al. (1998)
*T. fusca* DSM43793, BTA‐2, TfH	Kleeberg et al. (1998)
*T. fusca* DSM44342, TfH42_Cut1	Herrero Acero et al. (2011)
*T. fusca* (strain YX), WSH03‐11, Tfu_0883	Chen et al. (2008)
*T. fusca* (strain YX), WSH03‐11, Tfu_0882	Chen et al. (2008)
*T. fusca*, *Tf*Cut_2 (Cut2‐kw3)	Roth et al. (2014)
*T. fusca* NRRL B‐8184, Cut1	Hegde and Veeranki (2013)
*T. fusca* NRRL B‐8184, Cut2	Hegde and Veeranki (2013)
*T. cellulosilytica* DSM44535, Thc_Cut1	Herrero Acero et al. (2011)
*T. cellulosilytica* DSM44535, Thc_Cut2	Herrero Acero et al. (2011)
*T. alba* AHL119, Est119, est2	Kitadokoro et al. (2012)
*T. curvata* DSM43183, Tcur_1278	Wei et al. (2014)
*T. curvata* DSM43183, Tcur0390	Wei et al. (2014)
*T. halotolerans*, Thh_Est	Ribitsch et al. (2012)
*T. alba*, Est1 (Hydrolase 4)	Hu et al. (2010)
*Saccharomonospora (Thermoactinomyces) viridis* AHK190, Cut190	Kawai et al. (2014)
Bacilliodota	
*Bacillus subtilis* 4P3‐11, BsEstB	Ribitsch et al. (2011)
Bacteroidetes	
*Aequorivita sp. CIP111184*, *PET27*	Zhang et al. (2022b)
*Kaistella* (*Chryseobacterium*) *jeonii*, PET30	Zhang et al. (2022b)
Metagenome‐derived without a phylogenetic affiliation	
PHL‐7 affiliated with the Thermoanaerobacterales	Sonnendecker et al. (2022)
No obvious affiliation, PET2, lipIAF5‐2	Danso et al. (2018)
Eukarya	
*Pseudozyma (Candida) antarctica* lipase B, CalB	Ronkvist et al. (2009), Carniel et al. (2017)
*Fusarium solani*, FsC (f. sp. cucurbitae)	Ronkvist et al. (2009), Carniel et al. (2017)
*Thermomyces* (*Humicola*) insolens cutinase, HiC	Ronkvist et al. (2009), Carniel et al. (2017)
*(B) PUR ester bond‐active enzymes*	
Proteobacteria	
*Comamonas (Delftia) acidovorans* TB‐35, PudA	Nakajima‐Kambe et al. (1997)
*Pseudomonas chlororaphis*, lipase, PueA, PueB	Ruiz et al. (1999)
*Pseudomonas fluorescens*, esterase PulA	Howard and Blake (1998), Vega et al. (1999)
*Pseudomonas protegens* strain Pf‐5 *pueA* and *pueB*	Hung et al. (2016)
Actinobacteria	
LCC, leaf compost metagenome, higly simliar to HRB29 locus GBD22443	Schmidt et al. (2017)
*Thermobifida (Thermomonospora) fusca*, *TfCut_2 (Cut2‐kw3)*	Schmidt et al. (2017)
*Thermobifida (Thermomonospora) curvata*, DSM43183, *Tcur_1278*	Wei et al. (2014)
*Thermobifida (Thermomonospora) curvata*, DSM43183, Tcur0390	Wei et al. (2014)
*Rhodococcus equi* TB‐60, 45 kDa urethanase, purified	Akutsu‐Shigeno et al. (2006)
Eukaryotic hosts	
*Pestalotiopsis microspora*, lipase, 21 kDa hydrolase, purified	Russell et al. (2011)
*Candida rugosa*, lipase, Lip1–Lip5 isoenzymes, purified	Gautam et al. (2007)

In addition to these, 19 million tons of bio‐based polymers (“bioplastics”) are produced that are mainly based on biological and renewable resources. In this context, bioplastics have to be distinguished from biodegradable sorts of plastics (Rosenboom et al., 2022; Wei et al., 2020). Bio‐based polymers include natural rubber (NR), polybutylene adipate terephthalate [also referred to as poly(butylene adipate‐*co*‐terephthalate); PBAT], polylactic acid (PLA), polybutylene succinate (PBS), poly‐hydroxyalkanoate (PHA) with poly‐beta‐hydroxybutyrate (PHB) being the mostly produced variant, polyglycolic acid (PGA), polycaprolactone (PCL), polylactic‐co‐glycolic acid (PLGA; copolymer of PLA and PGA), poly(ethylene adipate) (PEA) and poly‐p‐dioxanone (PDS) [sorted by their global production capacity (European‐Bioplastics, 2022)]. Furthermore, starch and cellulose blends are among the main biopolymers. For many of the above‐named polymers, variants are available and alone for PHA, more than 150 are known (Li et al., 2016). All these polymers are already partly implemented in circular bioeconomy concepts and used at increasing, but still relatively low levels (Morell, 2019). It is assumed that most of these bioplastics are biodegradable like e.g. PLA, PHB, PCL and PBS (Narancic et al., 2018). This means that these polymers can be degraded over long time periods as defined by standard technologies and protocols (Vert et al., 2012). Regarding the produced amounts, NR lies at a level of 13 million tons per year, followed by PLA with annual amounts of approximately 0.4 million tons. All other bio‐based polymers are produced at less than 1 million ton annually (European‐Bioplastics, 2022). Notably, this equals less than 2% of the fossil‐fuel‐based polymers (Figure 1). Within this framework, it is noteworthy that biodegradable polymers like PHAs and PLAs are often produced using fossil resources (Figure 1). In fact, 40%–50% of the biodegradable polymers are based on fossil fuels (Statista.com, 2022).

Biodegradable polymers are in general used in the medical field for drug delivery and wound coverage, production of composting bags and packaging material. However, with the exception of rubber, none of the above‐mentioned biodegradable polymers has made it into high‐end applications and truly high‐level production scale compared to any of the fossil‐fuel‐based polymers (Choe et al., 2021; Di Bartolo et al., 2021; Raza et al., 2018; Zhao et al., 2020).

The global use of synthetic and fossil‐fuel‐derived polymers on a million‐ton scale, which has now been going on for more than 80 years, the lack of multinational concepts for re‐ and upcycling or circular use in combination with improper disposal have led to an unprecedented and seemingly mostly irreversible accumulation of plastics of various sizes and blends in almost all ecological niches (Bläsing & Amelung, 2018; Brandon et al., 2019; Geyer et al., 2017; Jambeck et al., 2015; Tekman et al., 2017). Thus, oceans, lakes, rivers, estuaries and soils harbour significant quantities of plastics at varying sizes. Based on these studies, it can be assumed that in the near future, smaller plastic particles (micro‐ and nanoplastics) will appear in all food chains and ultimately also have an impact on human and animal health and nutrition. In addition, an influence of microplastics on global biodiversity is to be expected (Hu et al., 2019; MacLeod et al., 2021; Zeytin et al., 2020).

In general, the particle sizes linked to the terms micro‐ and nanoplastics refer to particles with a diameter of 1 μm to 5 mm in case of microplastics, and for nanoplastics with a diameter of 1 nm up to a size of 1 μm. Currently, there is still some discussion about overlapping size ranges between nano‐ and microplastics and the upper size of microplastics (Gigault et al., 2018; Mitrano et al., 2021; ter Halle & Ghiglione, 2021).

In the light of the above made in part alarming reports, research in microbiology over the last decade has addressed the question, if and to which extent microorganisms can decompose fossil‐fuel‐derived plastics using enzymatic routes. While many recent reviews have addressed the phylogeny of microbial communities affiliated with the plastisphere and their global distribution, only few studies have indeed addressed the issues of enzymatic plastic breakdown and the phylogenetic distribution.

Therefore, within this review, we first point out a few often‐neglected aspects concerning microbial plastics degradation. Then, we summarize the status quo of microbial and enzyme‐driven degradation of the different sorts of artificial polymers. We further highlight recent advances and outline strategies used for finding novel plastic‐active enzymes. Lastly, we report on the usefulness of enzymes in plastic degradation on industrial scale and in nature.

## WEATHERING: PHYSICAL, MECHANICAL AND CHEMICAL DESTRUCTION OF PLASTICS IS AN ESSENTIAL AND FIRST STEP TOWARDS MICROBIAL DEGRADATION

**FIGURE 2:**
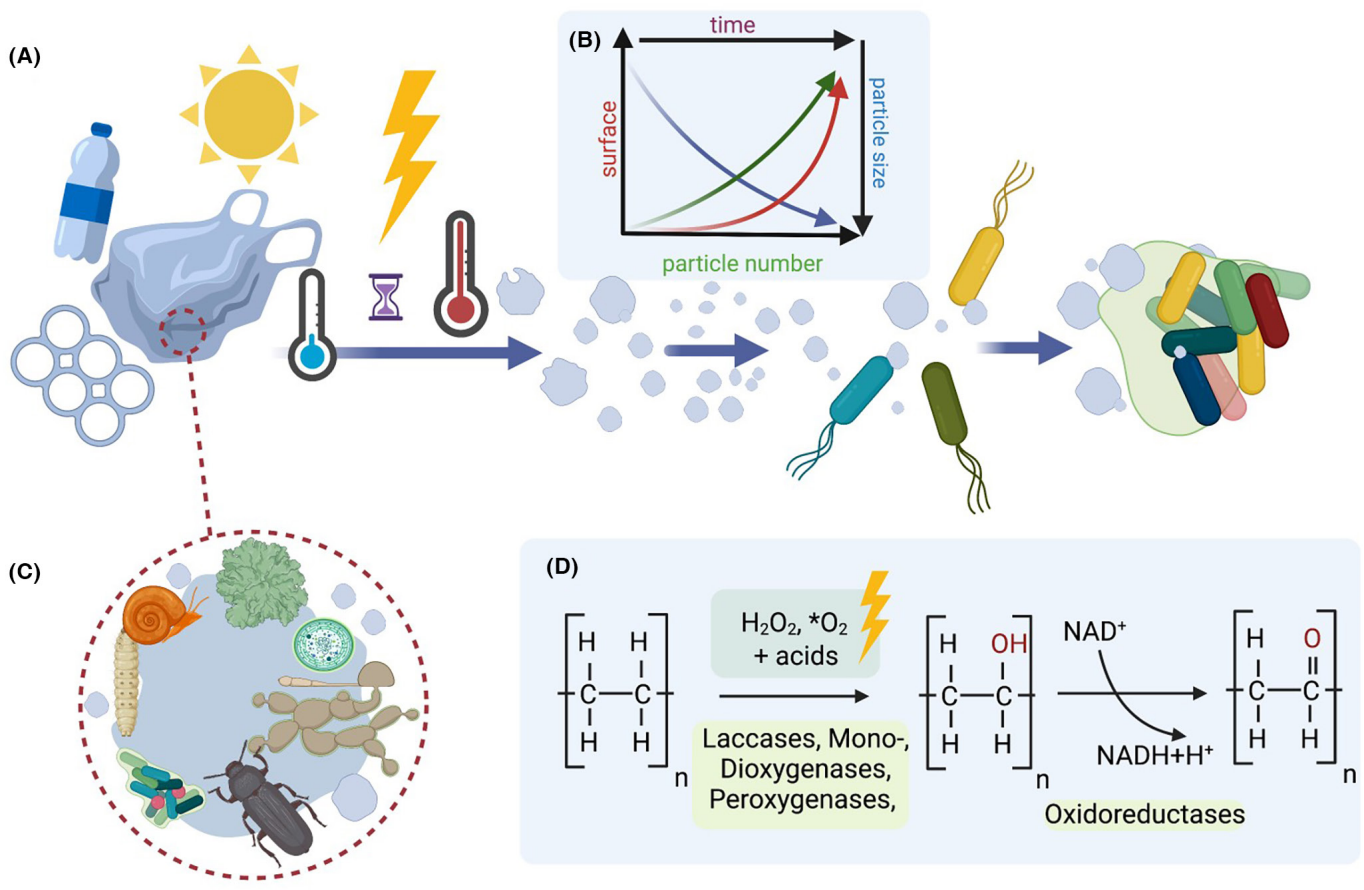
Weathering as an essential prerequisite for microbial colonization and enzymatic degradation of plastics. (A) The main abiotic factors are indicated and are mainly based on mechanical and physical destruction. (B) Increasing particle number results in decreasing particle size and strongly increasing overall surface of larger plastics broken down. (C) Insects, other animals, fungi and algae can be involved in biofouling and polymer disruption. Insects (worms, larvae) and snails eat through larger plastic particles of polystyrene (PS), polyethylene terephthalate (PET) and polyethylene (PE) (Bombelli et al., 2017; Song et al., 2020; Yang et al., 2015a). (D) Possible biotic and abiotic mechanisms as a part of biofouling. Random oxygenations can be involved in the hydroxylation olefins to break within the polymer at very low frequencies (Chamas et al., 2020; Norrish & Bamford, 1936).

As soon as larger plastic pieces (e.g. bags, bottles, fishing nets) are disposed into the environment, the weathering begins, which is a very slow process lasting decades, hundreds and thousands of years (Chamas et al., 2020; Duan et al., 2021). In nature, weathering is mainly caused by abiotic factors. Figure 2 summarizes many of the currently known physical and chemical processes involved in weathering. In the sea and on beaches, for example, the mechanical movement of the waves in combination with sand will cause a plastic bottle to be ground into smaller particles. In addition, ultraviolet (UV)‐radiation particularly attacks those polymers that contain aromatic compounds. This applies to PET in particular and to many plasticizers. Earlier studies have shown that UV‐light causes changes in the physical properties of PET, resulting in a colour change from clear to slightly yellow (Day & Wiles, 1972; Mohammadian et al., 1991; Shaw & Day, 1994). Furthermore, UV‐radiation also decreases the hydrophobicity of PET‐surfaces (Gotoh et al., 2011). Frequent temperature and pH changes also have an impact on material stability (Figure 2). Ester bonds may be affected by either very low pH‐values or the opposite. Abiotic weathering influences the polymers' degree of crystallinity. In case of semicrystalline polymers such as PE, it leads from a less ordered (amorphous) state to a well‐organized state (high crystallinity) to reach a thermodynamic equilibrium. Highly crystalline plastics are brittle and polymer chains break easily, thus, the fragmentation process into smaller particles begins. The consequences of this irreversible production of micro‐, nano‐ and even picoplastics with particle diameters ranging from milli‐ to picometers can be made clear by a sample calculation: The surface of a 500 ml standard plastic bottle with an area of approximately 950 cm^2^ will be increased by a factor of 1000, if broken down into particles with a diameter of 1 μm (Figure 2). If the same bottle is broken into pieces of 12 nm in diameter, the surface roughly increases to the average size of a soccer field with approx. 7140 m^2^. The generation of reduced particle sizes runs in parallel with increasing surface areas and altered hydrophobicity, thus allowing better microbial colonization and the beginning of the biotic process. Weathering will also contribute to the release of additives. For excellent reviews on mechanical, chemical and physical weathering as well as plastics stability, consult correspondent bibliography (Arp et al., 2021; Brandon et al., 2016; Duan et al., 2021; Gewert et al., 2015; Liu et al., 2020; Min et al., 2020; Sang et al., 2020).

As soon as microorganisms attach themselves to the polymers' surfaces, they possibly commence with a biotic degradation process. Consequently, secreted enzymes (hydrolases, C‐C‐lyases, oxidases, oxygenases, reductases etc.) can attach to the altered surfaces and single fibres. Without weathering, enzymatic degradation of the polymers in nature and in industries is hardly possible. In an industrial or laboratory scale, this process is simulated using thermal, UV‐light, pressure and/or mechanical pre‐treatments. However, it has been reported that under laboratory conditions no increase in PET degradation was observed after UV treatment (Falkenstein et al., 2020). For PE, which has no aromatic group, a UV‐driven oxidation may as well be an important process during weathering and can lead to the occurrence of Norrish type I and II reactions in which intramolecular γ‐H abstraction generates ketones and vinylidenes (Chamas et al., 2020; Norrish & Bamford, 1936).

Another case where the size of plastic particles is decisively changed in the environment and, especially in terrestrial sites, includes not only insects (larvae), but also snails that eat through larger plastic particles (PS, PET and PE) and thereby mechanically produce smaller particles (Bombelli et al., 2017; Song et al., 2020; Yang et al., 2015b). These particles can be ingested by worms and insects and further reduce in size. The effects of the gut microbiome on the polymer particles and vice versa are the subject of many recent studies (Brandon et al., 2018; Lou et al., 2020).

## MICROBIAL PLASTIC COLONIZATION IS NOT INDICATIVE FOR ENZYMATIC BREAKDOWN

The enlarged surfaces of micro‐ and nanoplastic particles allow an increased attachment and colonization by microorganisms in the form of a biofilm (Flemming et al., 2016). In nature, a mixture of different eu‐ and prokaryotic organisms colonize plastic particles (Amaral‐Zettler et al., 2020; Kirstein et al., 2019; Yang et al., 2020; Zettler et al., 2013). The plastic microbiota is termed the plastisphere and currently subjected to in‐depth studies. Notably, establishing a multispecies plastisphere does not necessarily mean polymer degradation is taking place. It is more likely that bacteria simply use the plastic particle as a surface and ecological niche and degrade chemical additives if they become available. Plastics colonization most likely depends on the chemical and physical properties of the polymer, the surface properties, the charge and the different types of additives present (Wright et al., 2021; Yang et al., 2020). Several studies have characterized the plastics microbiota. Bacteria often associated with marine plastics are affiliated with the *Nannocystaceae*, *Flavobacteriaceae*, *Planctomycetes*, *Saprospiraceae*, *Erythrobacteraceae*, *Hyphomonadaceae* and *Rhodobacteraceae* (Kirstein et al., 2019). There are first indications that bacteria affiliated with the genus *Vibrio* that are pathogenic to fish and humans accumulate noticeably frequently on the particles (Kirstein et al., 2016).

While the majority of these bacteria do not harbour enzymes to degrade any of the fossil‐fuel derived polymers, we speculate that they may have impact on the plastic stability and result in a very slow biofouling process. This is in our view partially caused by the release of organic acids produced by fermentation and different oxygen radicals within the plastic‐attached biofilms. It is well known that bacteria growing in biofilms produce and release significant amounts of organic acids (Flemming et al., 2016). In combination, the organic acids together with oxygen radicals (i.e. H_2_O_2_) can form peracid, which is a very strong oxidative agent toxic to bacteria but could play a key role in long‐chain alkane activation (Figure 2; Kiejza et al., 2021). By this, oxygen and OH groups would be introduced into the polymer backbone randomly and with very low frequency. This process would mainly concern the highly inert and stable C‐C‐bonds in olefins and it is a mechanism similar to the Norrish reactions (Chamas et al., 2020; Inderthal et al., 2021; Norrish & Bamford, 1936). The introduction of OH‐groups will eventually lead to the first break points within the otherwise inert polymeric C‐C‐chain (Figure 2). The latter are then the possible starting point for additional oxygenation and hydroxylation and possible breaks in the polymer. Involved enzymes will be mono‐ and dioxygenases, peroxygenases and laccases (Hofrichter et al., 2015; Mate & Alcalde, 2017). This type of plastic biofouling will run in parallel to the physical and chemical weathering outlined above and it will clearly take many years or even centuries to introduce sufficient break points in a polymer.

## ADDITIVES ARE THE PREFERRED CARBON SOURCES FOR MICROORGANISMS RATHER THAN THE POLYMERS

The requirements for additives differ from one plastic type to another. However, the majority of plastics contain additives. Commonly used additives are flame retardants, lubricants, plasticizers, antioxidants, acid scavengers, antistatic agents, biocidal agents, anti‐counterfeiting agents, light and heat stabilizers, colouring pigments, reinforcement fibres, slip compounds, thermal stabilizers and others (Carmen, 2021; Hahladakis et al., 2018). Their overall content can range from less than 0.1% to up to 70%. The repertoire of chemical compounds used for the additives differs widely. These are often not only organic compounds, but also different metal ions that can be part of the additives. Today, up to 10,000 different chemical compounds have been listed that can be considered as suitable additives (Groh et al., 2019; Wiesinger et al., 2021). For example, plasticizers are often esters of phthalic, trimellitic, benzoic and adipic acids. Bis(2‐ethylhexyl) phthalate (DEHP) and dibutyl phthalate (DBP) are most widely used.

Notably, there is growing concern on the toxicity of these additives, of which a large fraction is listed as substances of potential concern (Wiesinger et al., 2021). They can bioaccumulate, play a role as endocrine disruptors or have cancerogenic effects even at very low levels (Groh et al., 2019; Mathieu‐Denoncourt et al., 2015).

Interestingly, the vast majority of the organic and non‐polymeric additives appear to be biodegradable over time by many soil and water‐borne microorganisms (Wright et al., 2020). For example, many of the plasticizers are phthalate esters that can be degraded under aerobic and anaerobic conditions (Boll et al., 2020). Under aerobic conditions, microorganisms employ dioxygenases to introduce hydroxyl functionalities into the phthalate esters and to facilitate subsequent decarboxylation. This is accomplished by employing either aromatizing dehydrogenases or cofactor‐free decarboxylases. Under anaerobic conditions, the phthalate esters are converted into thioesters using coenzyme A (CoA)‐ligases or CoA‐transferases prior to a decarboxylation step (Boll et al., 2020).

## ENZYMATIC DEGRADATION OF FOSSIL‐FUEL‐DERIVED PLASTICS IS LIMITED TO PET, PUR AND FEW PA OLIGOMERS

While PET, PA and ester‐based PUR have enzymatically hydrolysable ester or amide bonds, the bonds of PE, PS, EP, PP and ether‐based PUR are much more difficult to cleave. For example, as a possible point of attack, an oxygen or hydroxylation must first be incorporated into the stable carbon chains and, in addition, PVC must be dechlorinated before any biodegradation can take place. Thus, any enzymatic depolymerization strategy must consider that different types of chemical bonds – with different strengths – need to be broken (Krueger et al., 2015b). Furthermore, a major challenge lies in the accessibility of the polymer chains, their surface charge and hydrophobicity or wettability. These factors can negatively affect enzymatic and microbial attachment. Another challenge hampering microbial degradation is that the polymers cannot be taken up into the cells. The cleaving enzymes need to be secreted and in case of oxidation processes, electrons and cofactors have to be present for the enzymes outside of the cell. For biopolymers like cellulose or starch, nature has evolved binding modules that assist the hydrolases in getting closer to the polymer (Lynd et al., 2002). No such enzymes are known to be involved in synthetic polymer degradation. In addition, no expansins and loosenin‐like proteins are yet known to act on synthetic polymers. These proteins loosen cellulose microfibrils, possibly through the rupture of intramolecular hydrogen bonds (Quiroz‐Castañeda et al., 2011; Sampedro & Cosgrove, 2005).

Furthermore, the degree of polymerization crystallinity has a strong impact on any enzymatic polymer breakdown. Today, no plastic‐active enzymes are known to act on highly crystalline synthetic polymers. Finally, water solubility of the polymers and their breakdown products will have a major impact on the possibility of enzymes to act on the fibres (Barth et al., 2015).

Despite these many challenges and obstacles, a total of close to 110 verified enzymes are listed in the Plastic‐Active Enzyme (PAZy) and the PlasticDB databases today, of which 38 act on PET, 11 on ester‐based PUR, further 11 on PA oligomers, 4 on NR, 22 on PLA and 15 on PHAs. No enzymes are known to act on PP, PE, PVC, PA polymers, ether‐based PUR and PS (Figure 1). These polymers have in common that they lack ester bonds but mainly consist of C‐C‐, C‐N‐ and ether‐bonds.

In the following, we give a short overview on the available polymer‐active enzymes. A detailed list of enzymes acting of fossil‐fuel based low‐ and medium‐density polymers is given in Table 1. Furthermore, an up‐to‐date list of plastic‐active enzymes can be retrieved from the PAZy database [www.pazy.eu (Buchholz et al., 2022)].

### PET‐active enzymes

With respect to the fossil‐based polymers, PET degradation is already quite well understood.

PET‐degrading enzymes belong to the classes of cutinases [EC (enzyme category) 3.1.1.74], lipases (EC 3.1.1.3) or carboxylesterases (EC 3.1.1.1) and these can only hydrolyse amorphous and low‐crystalline PET. The PET‐active enzymes (PETases) hydrolyse the ester bond to produce either bis‐(2‐hydroxyethyl) terephthalate (BHET), mono‐(2‐hydroxyethyl) terephthalate (MHET), or terephthalic acid (TPA) and ethylene glycol (EG). It is not yet clear, if the enzymes act as endo‐ or exo‐enzymes (Danso et al., 2019; Taniguchi et al., 2019; Wei & Zimmermann, 2017; Wright et al., 2021). MHET can then be cleaved with a specific mono‐(2‐hydroxyethyl) terephthalate hydrolase (MHETase) and the TPA monomers are degraded by cleavage of the aromatic ring structure via known aryl degradation pathways yielding protocatechuic acid. The enzymes involved in this step are mono‐ and dioxygenases observed in *Comamonas testosteroni*, *Delftia tsuruhatensis*, *Rhodococcus* sp., *Pseudomonas umsongensis*, *Acinetobacter baylyi* and others (Choi et al., 2005; Kincannon et al., 2022; Narancic et al., 2021; Schläfli et al., 1994; Wang et al., 1995). EG is metabolized using the glyoxylate carboligase pathway (Li et al., 2019).

The known PET‐active enzymes originate from four bacterial and two fungal phyla (Table 1, Zhang et al., 2022a). No archaeal enzymes have been reported acting on PET. Notably, few of the PET‐active enzymes are also acting on ester‐based PUR (Schmidt et al., 2017). Many of the PET‐active enzymes are thermostable and perform best at temperatures between 55 and 65°C. This temperature is close to the glass transition temperature range of PET (65–71°C) where harder and brittle polymer domains turn softer, more flexible and thus enzyme‐accessible. Protein engineering is therefore aimed at creating PETases with higher thermostability and improved catalytic efficiency at elevated temperatures (Wei et al., 2022; see also “5. Synthetic approaches to obtain novel or improved enzymes”). Few enzymes degrade PET at lower temperatures, implying they may play a role in colder climate zones (Zhang et al., 2022b). However, all known native PET‐active hydrolases have a rather low catalytic activity towards high‐molecular‐weight PET and all are promiscuous enzymes, implying that PET is not the native substrate. Notably, esterases are well known to be promiscuous enzymes. Few of such enzymes are known to turn over more than 70 different chemical substrates (Leveson‐Gower et al., 2019; Martinez‐Martinez et al., 2018).

**FIGURE 3:**
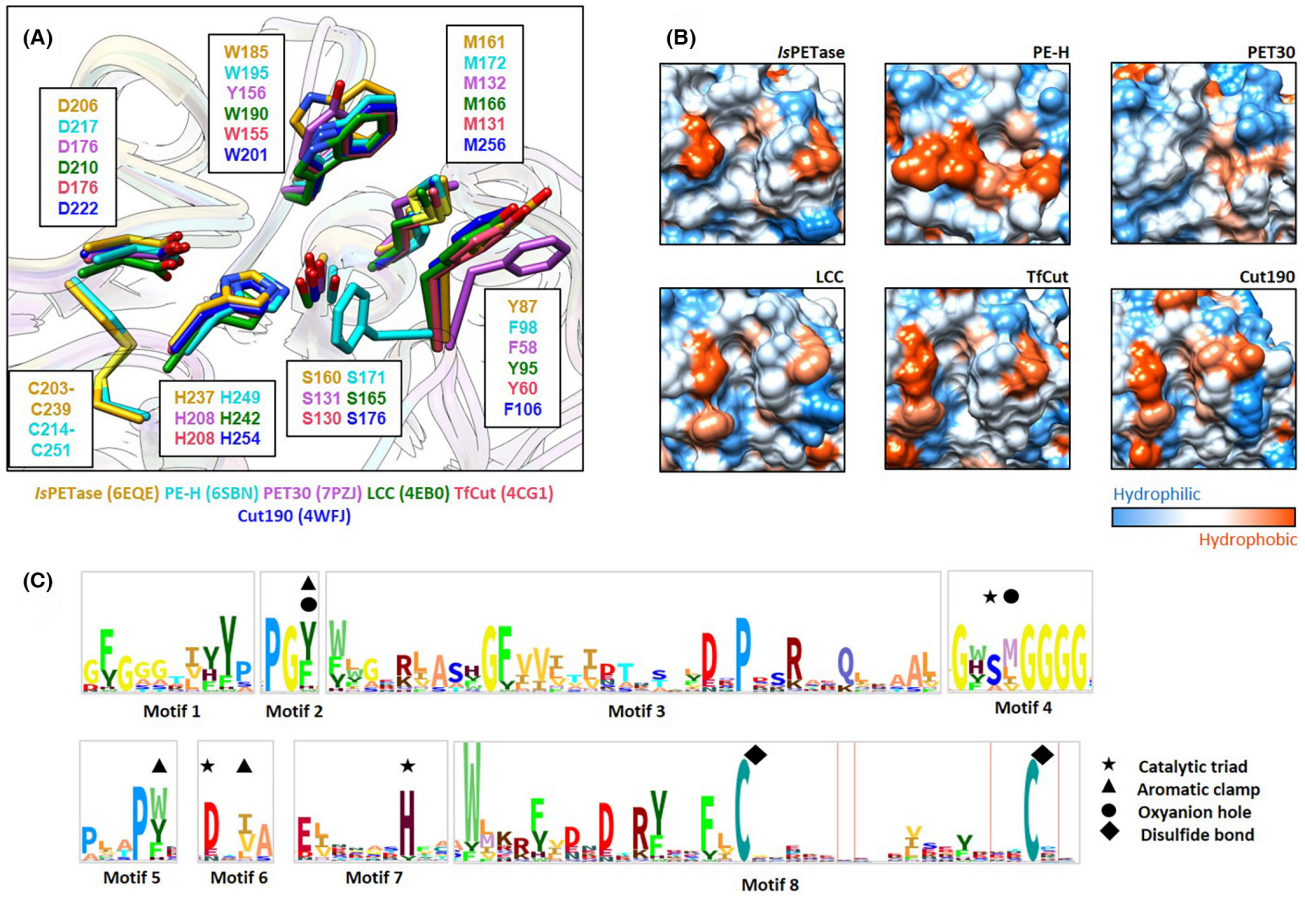
Common structural traits of polyethylene terephthalate (PET)‐active enzymes. The depicted structures are based on *Ideonella sakaiensis* PET hydrolase (*Is*PETase) (PDB code 6EQE), PE‐H (6SBN), PET30 (7PZJ), LCC S165A* (6THS), *Tf*Cut (4CG1) and Cut190 (4WFJ). (A) Overlay of the catalytic amino acids within the active sites involved in PET‐degradation. (B) Surface representation of the enzymes leading to the active site cavities. Hydrophobic residues play an important role in the access of the polymers into the catalytic site. (C) The Hidden Markov Model (HMM) motif shows the conserved amino acid patterns of known PET‐active enzymes (PETases) and was derived from Danso et al. (2018).

Still, they are often‐designated PETases. While PET esterases are not highly conserved among each other, few structural traits and sequence homologies appear to be common in most of the known enzymes (Figure 3). Based on our data analyses and others (Wei et al., 2022), it becomes evident that none of the current enzymes carries a lid domain. All active enzymes are secreted proteins, carry at least an N‐terminal signal peptide and some even a PorC‐like type 9 secretion system affiliated signal (Zhang et al., 2022b). The region involved in substrate binding contains in general the amino acids T/F–M–W/T and the catalytic triad is composed of D–H–S. Furthermore, active enzymes carry 1–2 disulphide bonds and of these, one is close to the active site. The active site is well accessible for the bulky substrates and is located in a rather large cavity. For a more detailed analyses of common PETase features, we refer to Wei et al. (2022).

The first PET‐active enzymes were indeed published already in 1998 and derived from the Gram‐positive *Thermobifida fusca* (Kleeberg et al., 1998, 2005) and the complete degradation pathway for PET was first described in the bacterium *Ideonella sakaiensis* (Yoshida et al., 2016). Today, the best‐studied enzymes are the PET‐hydrolase derived from *I. sakaiensis*, the above‐named *T. fusca*‐derived enzymes and the metagenomic leaf compost cutinase (LCC) (Hiraga et al., 2019; Taniguchi et al., 2019). The latter was originally derived from leaf compost, but based on sequence similarities, it is most likely affiliated with the Actinobacterial strain HRB29 (Sulaiman et al., 2012; Xi et al., 2021). Only few other enzymes are studied in detail. Among those, a number of actinobacterial enzymes like TFcut_2 (Roth et al., 2014), the Bacteroidetes enzyme PET30 (Zhang et al., 2022b) and few *Pseudomonas*‐derived enzymes (Bollinger et al., 2020; Haernvall et al., 2017b; Wallace et al., 2017).

**FIGURE 4:**
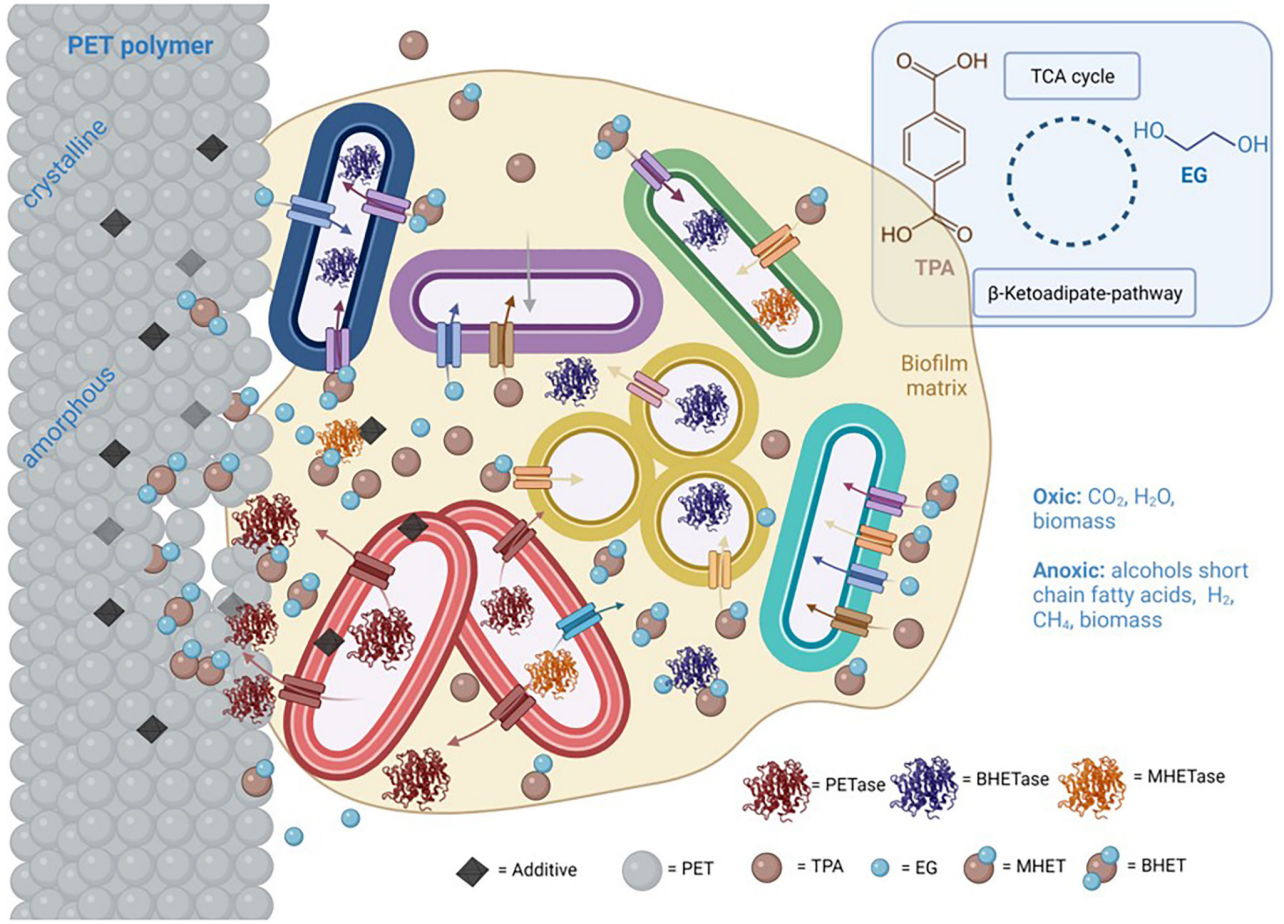
Model for enzymatic degradation of polyethylene terephthalate (PET) by mixed and multispecies microbial consortia. Enzymatic degradation occurs only at low‐crystalline regions of the polymer. The presence of bis‐(2‐hydroxyethyl) terephthalate hydrolases (BHETases) and mono‐(2‐hydroxyethyl) terephthalate hydrolases (MHETases) is optional and depends on the organisms.

While it has not been shown that PET can be degraded in native environments by any of the known PETase producers, it is likely that PET degradation would be accomplished as a community job by mixed species microbial consortia. Figure 4 summarizes the essential steps of the enzymatic PET‐degradation resulting in TPA and EG. Some organisms produce PET‐hydrolases, but others are taking up EG and TPA. In this context, it is worth noting that some organisms can absorb and metabolize TPA. These include, for example, *Burkholderia xenovorans*, *Comamonas testosteroni* and *Comamonas thiooxidans*. These bacteria probably benefit from the presence of a PET‐active organism in the vicinity. In *C. thiooxidans*, for example, a tripartite tricarboxylate transporter (consisting of the membrane proteins TpiA, TpiB and the substrate‐binding protein TphC) is responsible for TPA transport into the cell where TPA is further degraded via protocatechuate (Chain et al., 2006; Hosaka et al., 2013; Kasai et al., 2010).

### PUR (ester)‐active enzymes

Ester‐based PUR can be degraded by very few microbes. However, the ether‐based forms cannot be degraded. For the enzymatic degradation of PUR, in total 12 verified and characterized active enzymes are available. They are derived from the phylum of the Proteobacteria (Howard & Blake, 1998; Hung et al., 2016; Nakajima‐Kambe et al., 1995, 1997), the Actinobacteria (Akutsu et al., 1998; Schmidt et al., 2017) and two fungal enzymes are known (Gautam et al., 2007; Russell et al., 2011). The enzymes belong to the cutinases (EC 3.1.1.74), lipases (EC 3.1.1.3) and carboxylesterases (EC 3.1.1.1) and are often referred to as polyurethanases (PURases) (Danso et al., 2019; Wei & Zimmermann, 2017). The most frequently used model substrate is Impranil (DLN). We further refer to the review by Liu *e*t al. (2021a) for a more detailed insight into PUR‐active microbial consortia and other useful background information.

### PA‐oligomer‐active enzymes

Similar to some of the above‐mentioned polymers, neither microorganisms nor enzymes are known, which are able to degrade the intact high‐molecular‐weight PA‐polymer. Few studies present convincing evidence that bacteria act on either linear or cyclic nylon oligomers with rather short‐chain lengths. These enzymes were isolated from various *Pseudomonas*‐species, *Paenarthrobacter ureafaciens*, *Agromyces* sp., *Kocuria* sp., *Nocardia* sp. and *Pseudoxanthomonas* sp. (Guo et al., 2013; Heumann et al., 2009; Kanagawa et al., 1989, 1993; Kinoshita et al., 1975, 1977; Negoro et al., 1992; Prijambada et al., 1995; Sasanami et al., 2022; Yasuhira et al., 2007; Yoshioka et al., 1991).

The biochemically characterized PA‐oligomer hydrolases are NylA, which is a 6‐aminohexanoate cyclic dimer hydrolase (amidase). NylB, which is a linear 6‐aminohexanoate dimer hydrolase and NylC, which hydrolyses linear trimers, tetramers and pentamers of 6‐aminohexanoate by an endo‐type reaction (Guo et al., 2013; Kanagawa et al., 1989, 1993; Kinoshita et al., 1975, 1977; Negoro et al., 1992).

### PBAT‐degrading enzymes

Among the biodegradable plastics, PBAT has one of the highest shares in global production (European‐Bioplastics, 2022). It is mainly used as foil for packaging and in agriculture as it has similar properties like low‐density PE. The co‐aliphatic–aromatic polyester is produced by a random polymerization of terephthalic acid (TPA) and adipic acid with 1,4‐butanediol. Its aliphatic components make it easier to be biodegraded by microbial enzymes belonging to the group of carboxylesterases, lipases and cutinases (pazy.eu). Examples are the esterase PpEst from *Pseudomonas pseudoalcaligenes* (Wallace et al., 2017), the lipase PfL1 from *Pelosinus fermentans* (Biundo et al., 2016) and the thermophilic hydrolase TfH from *Thermobifida fusca* (Kleeberg et al., 2005).

### PHA‐active depolymerases

Since PHAs are considered “natural polymers”, their degradation is in principle possible. The biodegradation of various sorts of PHAs has been observed not only in many different bacteria but also in fungi from marine and terrestrial niches (Jendrossek & Handrick, 2002; Suzuki et al., 2021; Viljakainen & Hug, 2021). The characterized and known enzymes involved are either secreted extracellular PHA (e‐PHA) or internal PHA (i‐PHA) depolymerases. *P. putida* KT2442´s PhaZ serves as the prototype of intracellular medium chain length polyhdroxyalkanoate (mcl‐PHA) depolymerases (De Eugenio et al., 2007, 2008). The biochemically best characterized enzymes are derived from the Gram‐negative bacteria *Cupriavidus necator* (*Ralstonia eutropha*) and *Paucimonas lemoignei*. Both are model organisms for PHA (PHB) metabolism and have been intensively studied. *C. necator* codes for six internal depolymerases, two external acting enzymes and two oligomer‐active enzymes. The well‐known depolymerases are classified as carboxylesterases (EC 3.1.1.75/EC 3.1.1.76). Notably, direct measurements of enzyme activities are technically very challenging (Jendrossek, 2007). A comprehensive list of characterized PHA‐degrading enzymes can be found in (Urbanek et al., 2020).

### Enzymes active on PLA

Polylactic acid (PLA, [–C(CH_3_)HC(=O)O–]_*n*_) (PLA) is one of the synthetic polymers that can be built using renewable sources (D‐, L‐lactic acid). PLA can be degraded by a wider range of bacteria employing esterases and serine proteases. The enzymatic degradation is rather slow and is depending on the degree of crystallinity, the type of the copolymer and the additives (Zaaba & Jaafar, 2020). Surprisingly, only 24 enzymes have been biochemically characterized in detail. They are derived from bacteria and very few from fungi and belong to the enzyme families of proteases, esterases/lipases, cutinases and depolymerases. The bacterial enzymes are mainly affiliated with the genera of *Amycolatopsis*, *Paenibacillus*, *Lederbergia*, *Alcanivorax* (Hajighasemi et al., 2016; Li et al., 2008; Oda et al., 2000; Sukkhum et al., 2009) and the fungal ones originate from *Parengyodontium*, *Aspergillus* and *Cryptococcus* (Masaki et al., 2005; Oda et al., 2000; Yamashita et al., 2005). In addition, few metagenome‐derived enzymes have been identified (Mayumi et al., 2008; Tchigvintsev et al., 2015). For a complete list of all known verified PLA‐active enzymes, we refer to the PAZy database.

### Oxygenases and dioxygenases involved in NR breakdown

Microorganisms capable of degrading NR are affiliated with the bacterial genera of *Mycobacterium*, *Gordonia*, *Nocardia*, *Steroidobacter*, *Streptomyces*, *Rhodococcus*, *Actinoplanes*, *Micromonospora* and others (Rose & Steinbüchel, 2005; Sharma et al., 2018; Watcharakul et al., 2016). In addition, several NR‐active enrichment cultures were published recently (Nguyen et al., 2020).

Despite the impression that the capability of microorganisms to degrade NR appears to be relatively widespread, only few enzymes have been characterized in more detail. Oxidative enzymes cleaving the double bonds within the isoprene polymers initiate NR‐degradation. The best‐studied enzymes include Lcp from *Streptomyces* sp. K30, which is a latex clearing protein encoded together with two other oxidases, OxiA and OxiB. Lcp is a b‐type cytochrome, and it acts as endo‐type dioxygenase. It generates oligo‐isoprenoids with a chain length of C20 and above that differ in the number of isoprene units, but have the same terminal functions, CHO‐CH_2_– and –CH_2_‐COCH_3_ (Birke et al., 2015; Ilcu et al., 2017; Jendrossek & Birke, 2019).

The RoxA protein is a (rubber) dioxygenase and it has two c‐type haem centres. RoxA was derived from *Steroidobacter cummioxidans* (*Xanthomonas* sp.) strain 35Y and it catalyses an oxidative C‐C‐cleavage. Notably, RoxB (LatA) is another type of rubber oxygenase from the same organism (Jendrossek & Birke, 2019).

### Lack of enzymes for PE, PP, PS, PVC, PA and ether‐based PUR

Since C‐C‐ and ether‐bond cleavages as well as dehalogenations are rather difficult enzymatic reactions, for none of the fossil‐fuel‐derived polymers PE, PP, PS, PVC, PA and PUR (ether‐based) biochemically characterized and verified enzymes are known that truly attack the polymers.

Some largely overinterpreted findings on enzymes acting on the above‐listed polymers had been reported recently, but were in general not supported by biochemical and sophisticated analytical data. This topic has also been addressed in a recent review concerning PE (Ghatge et al., 2020) and other polymers. For PE, growing evidence suggest that alkane monoxygenases and alcohol dehydrogenases may possibly play a crucial role in larger C‐C‐chain degradation. It had been reported earlier that a predicted alkane hydroxylase, AlkB, was able to break down low‐density and low‐molecular‐weight PE [LMWPE (Yoon et al., 2012)]. AlkB is a predicted alkane monooxygenase with a multicopper domain similar to a laccase (Jeon & Kim, 2015). Therefore, it is highly likely that AlkB or homologues are involved in the initial oxidation of long‐chain alkanes. However, this study falls short of providing convincing analytical data clearly demonstrating the release of PE oligomers. Given the nature of the LMWPE that had been self‐prepared, and the indirect evidence provided by these authors, it is more likely that AlkB converted long‐chain alkanoates present in the PE preparations or fatty acid residues from the membranes of bacteria after cell lysis (Yoon et al., 2012). Similar reports on *Bacillus* and *Paenibacillus* isolates acting on PE also fell short of providing convincing data on the PE‐degradation products of the postulated AlkB enzymes' involvement in PE‐degradation (Bardají et al., 2019; Yang et al., 2015a). Within these settings, one of the first reports on possible PE‐active microorganisms identified the fungal isolate *Penicillium* sp. YK as a potential PE‐degrader (Yamada‐Onodera et al., 2001). The study reported that the fungus significantly reduced the molecular weight of the polymer after incubation of over three months. In a more recent follow‐up publication, the first enzymes possibly involved were identified as a laccase and a manganese peroxidase (Sowmya et al., 2015). Unfortunately, no biochemical characterization was performed, and no protein sequence was deposited. However, the recent study is so far the only one that linked enzymatic activities to high‐molecular‐weight PE‐degradation. The possible involvement in the initial breakdown of long‐chain alkanes was further supported by transcriptome studies using *Rhodobacter opacus* R7 (Zampolli et al., 2021). *Rhodobacter opacus* R7 grown on PE and RNA sequencing (RNAseq) analyses implied that at least one multicopper oxidase was 19.5‐fold induced under these conditions. Similarly, a study describing a copper‐dependent laccase from *Rhodococcus ruber* potentially being involved in PE degradation fails to provide conclusive evidence supporting enzymatic turnover of PE (Santo et al., 2013).

While most reports on PE‐degrading bacteria address isolated bacteria from soil or marine communities, few studies linked PE‐degrading microbes with the gut microbiome of wax worms (Bombelli et al., 2017; Cassone et al., 2020; Yang et al., 2014, 2015a). The studies provided first evidence on wax worms, which are able to grind the PE foil mechanically and on the microbiota of PE‐fed larvae adapting to this special diet. Unfortunately, in none of these promising studies defined PE‐cleaving enzymes were reported.

Similarly, no enzymes have been characterized that act on PS‐polymers. The degradation of the monomer had been well studied and bacteria like *Alkanivorax borkumensis* harbour the required genes and enzymes to hydroxylate the aromatic ring in the styrene monomers (Schneiker et al., 2006; Yakimov et al., 1998). For an excellent review on styrene degradation, see (Mooney et al., 2006). Recently, it had been reported that *Acinetobacter johnsonii* JNU01 and *Pseudomonas lini* JNU01 would be able to degrade polymeric PS. The enzymes presumably linked to the degradation are AlkB and AdhH homologues (Kim et al., 2021). PS biodegradation had also been reported for the microbiota of mealworms and other insects (Brandon et al., 2021; Kim et al., 2020; Peng et al., 2019; Yang et al., 2015b). These promising and surely very innovative studies, however, failed to provide convincing data showing the degradation products and did not provide insight into a possible enzyme's involvement. A very recent study even attempted to increase the assumed plastic biodegradation rates by enhancing gut microbiome‐derived enrichments outside the gut microenvironment of mealworms (Brandon et al., 2021). Unfortunately, the data provided in this innovative study did not support the concept that the mealworm microbiota is capable of digesting PS polymers.

Similarly, multiple enrichment cultures, single isolates and also microbiota‐based studies have been published claiming the possible degradation of PP (Arkatkar et al., 2010; Helen et al., 2017; Jain et al., 2018) and PVC (Giacomucci et al., 2019; Kırbaş et al., 1999; Peng et al., 2020). While these and other studies in this field are very promising and helped to enrich plastic‐affiliated biodiversity, they have not been successful in identifying defined enzymes acting on any of the polymers.

## SMART STRATEGIES FOR THE IDENTIFICATION OF PLASTIC‐ACTIVE ENZYMES

Finding enzymes acting on PE, PP, PS, PA, PVC or ether‐based PUR is a very challenging task that needs to be addressed through combined efforts of well‐trained microbiologists, bioinformaticians and analytical experts providing the right tools to verify the actual degradation with great care and accuracy.

A detailed search in PubMed (April 2022) using the key words “microbial plastic degradation” identified 7400 entries for studies on plastic‐active microorganisms. Most of these publications used enrichment strategies combined with weight loss as an indicator of microbial degradation. Others have studied surface alterations using scanning electron microscopy (SEM) and/or Fourier transform infrared (FTIR) technologies. Since weight loss also indicates the degradation of additives, it is likely that in many of these studies bacteria had been enriched that consumed the additives rather than the polymer. This would also apply in part to the studies that monitored changes in the surface and this would explain why the final proof of polymer degradation is often missing and why many studies have failed to provide active enzymes.

In summary, these studies are certainly very valuable and resulted in the accumulation of a rich and useful biodiversity. At the same time, they teach us that these are very rare and difficult‐to‐find enzymes.

### What stumbling blocks are there to be considered?

Recently, another type of study appeared in PubMed solely relying on bioinformatic and literature‐based searches for plastic‐active enzymes. One of these studies mainly relied on keyword searches (Gambarini et al., 2021). While these studies certainly give an overview on the current field, simple keyword searches do not capture all studies. Furthermore, they deliver mainly predicted enzymes and bear a significant risk of false positives being included. This can be explained in part by the unintentional, but unfiltered use of incorrectly annotated GenBank entries and/or the failure to carefully inspect the obtained references applying strictly analytical and biochemical parameters. In at least one case, this has already led to global distribution models for PE‐ and PS‐acting enzymes, despite the notion that the enzymes are still not known for these polymers (Zrimec et al., 2021). The same study also largely predicted the involvement of non‐secreted enzymes. Thus, non‐critical use of potential plastic‐degrading gene sequences ultimately leads to incorrect models and global distribution patterns of these enzymes and their role in nature. These studies will mislead researchers, environmentalists, policy‐ and lawmakers and even the broader public audience by implying that we have enzymes at our hands for solving the global plastics crisis, which we, however, do not have.

Within this framework, it is questionable, if it is possible to cleave C‐C bonds in larger polymers at all using enzymatic processes. Calculations on the free energy needed to crack the C‐C bonds and other challenges linked to polymer degradation imply that it might be almost impossible to establish enzymatic processes for many of the olefins (Jiang et al., 2021; Krueger et al., 2015a).

### Which strategies are best suited to identify novel plastic‐active enzymes?

Looking at the need to identify plastic‐active enzymes, several strategies should be applied.

#### Enrichments, from landfill and garbage sites but also soils and compost

Habitats that both contain complex polymeric substrates and a rich diversity and high abundance of microorganisms seem to be promising sources for new degradative enzymes (Bardají et al., 2019; Hu et al., 2010; Sulaiman et al., 2012). Despite the above‐mentioned challenges enrichment technologies bear, the use of these is still very timely and appropriate, especially given the possibilities to use enrichments in combination with robotics and AI approaches. Large numbers of enrichment samples could be processed by testing a very wide range of environmental parameters to enrich for difficult or almost non‐culturable bacteria. In combination with omics‐analyses and sophisticated analytical technologies, smart enrichments can deliver polymer‐active enzymes. Using RNAseq, metabolomics and proteomics in these multispecies consortia will further give us clues on key genes involved in polymer degradation (Tesei et al., 2020; Zampolli et al., 2021).

#### Metagenome mining as a rich source for novel enzymes

In addition to enrichment strategies, the use of classical metagenome mining approaches is certainly a very useful strategy. Few plastic‐active enzymes have been identified using metagenome approaches, although it has been noticed that the enzymes are not very abundant (Danso et al., 2018; Sonnendecker et al., 2022). Two strategies are possible: a sequence‐based approach and a functional approach. The functional approach will use small or large insert libraries in heterologous hosts in combination with analytical technologies (Ferrer et al., 2016). In order to find these rare enzymes, correspondingly large quantities of highly diverse metagenomic DNA from promising environments would have to be stored in the libraries to increase the chances of success. Another hurdle is the sometimes insufficient heterologous production of the enzymes of interest and rather high detection limits. To foster the metagenomic approach, the development of direct, rapid and efficient screening methods with specific plastic‐related substrates would need to be advanced. Functional gene mining is certainly a very challenging task, but it would clearly deliver novel enzyme diversity. Notably, the functional searches should be combined with high‐throughput technologies and smart screening systems.

#### Gene mining in databases using Hidden Markov Models and structural motifs.

Mining through large genome databases and using homology‐based approaches is certainly a straightforward approach. Thereby the best results have been obtained by using well‐designed and experimentally verified profile Hidden Markov Models (HMMs). These searches have been applied very successfully based on already known and functionally tested enzymes in a recent study (Danso et al., 2018). The design and experimental verification of the HMM motifs is, however, mandatory. Since esterases are in general a phylogenetically highly heterogenous enzyme family, failure of using not experimentally verified HMM profiles bears the risk of identifying false positives. A recent study in which HMMs were used based on only two sequences largely predicted false positives for a wide range of potential plastic‐active enzymes and also not considering that the enzymes should be secreted (Zrimec et al., 2021).

It can be expected that searches will be improved by switching from0 HMM‐only searches to structure‐based searches and by relying on global structure prediction tools like AlphaFold2 or the Robetta server. For more details, we refer to the webpages of the respective tools at https://github.com/deepmind/alphafold and at https://robetta.bakerlab.org.

**FIGURE 5:**
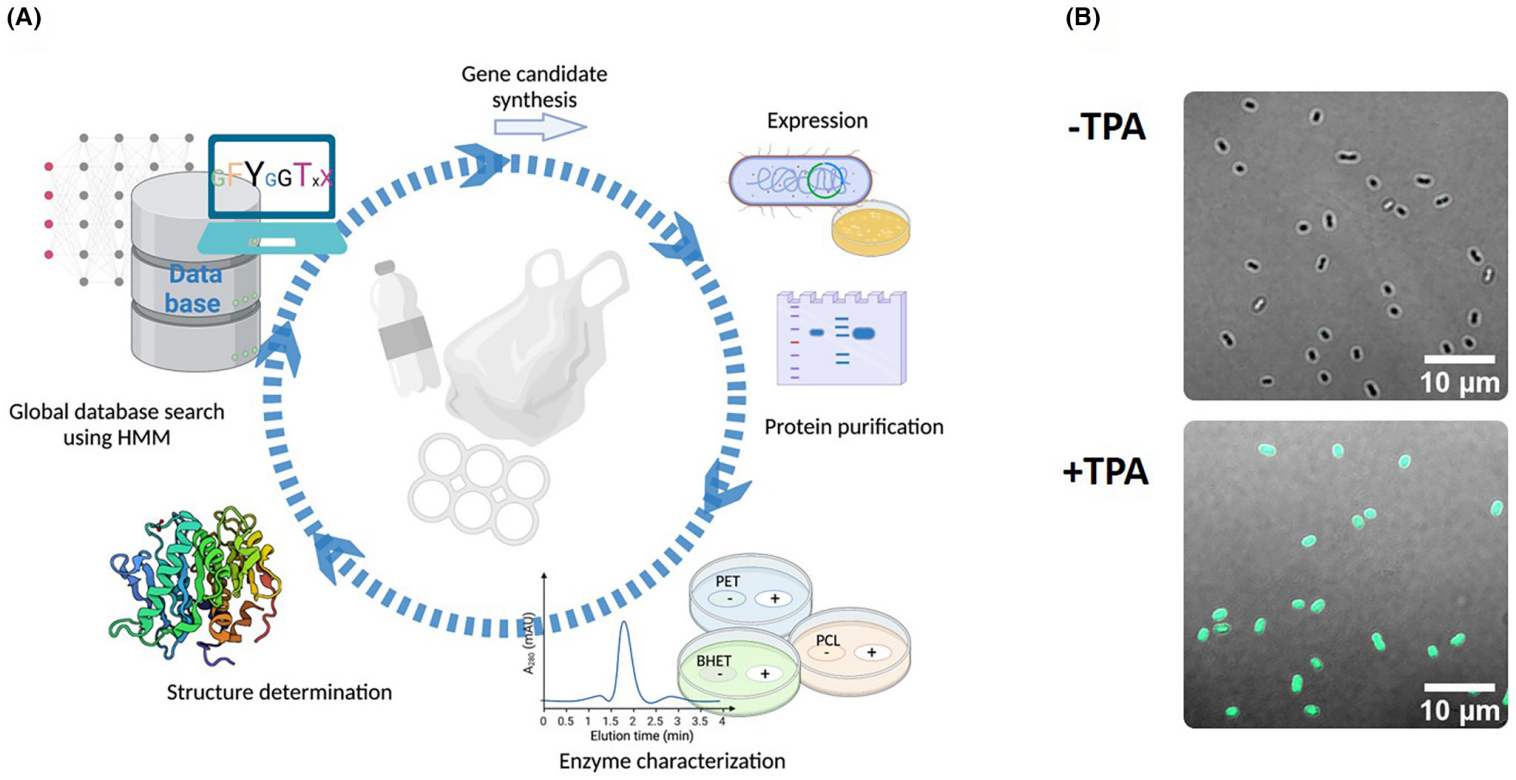
Strategies for identification of novel plastic‐active enzymes. (A) Using Hidden Markov Model (HMM)‐based searches in combination with in vitro transcription and translation of targets can be one of the fastest strategies to obtain novel enzymes. (B) Using reporter strains that are able to detect terephthalic acid (TPA) uptake can be a highly sensitive way to detect polyethylene terephthalate (PET)‐degrading activity. Image shows a recombinant *Comamonas* sp. strain E6 carrying a *thpA:sfGFP* (tetrahydrophthalic anhydride/superfolding variant of green fluorescent protein (GFP)) promoter fusion in the presence or absence of 50 μm TPA. Images contain unpublished data from our laboratory.

As recently outlined, the use of in vitro technologies in combination with HMM‐based screening will deliver quickly new biodiversity (Figure 5; Danso et al., 2018, Markel et al., 2020). By applying in vitro transcription and translation technologies, the time‐consuming protein production in heterologous hosts can be circumvented for the first rounds of activity screening.

Within this framework, having well‐curated databases on plastic‐active enzymes available is certainly an essential key to the successful identification of truly active enzymes. Today, three databases have picked up this topic. They are designated PMBD (Plastics Microbial Biodegradation Database) (Gan & Zhang, 2019), PlasticDB (Gambarini et al., 2022) and PAZy (Buchholz et al., 2022). All databases give a current overview on potential and verified enzymes acting on plastics. However, all databases have a slightly different focus and data contents. While PAZy contains exclusively functionally verified and manually curated enzymes, PMBD and PlasticDB contain larger data sets of predicted enzymes and microorganisms detected in enrichment cultures. A major challenge lies, however, in the interpretation of the results produced by the different databases. While a search for PE‐ or PS‐active enzymes results in no hits in PAZy, the same search in PMBD and PlasticDB identifies non‐verified hits. Notably, in PlasticDB, the predicted ones are clearly labelled as such.

#### Developing ultrasensitive biosensors for degradation products

Today, several other methods are known to assess the degradation of PET by candidate enzymes. These methods are mostly based on the direct or indirect detection of the breakdown products TPA, MHET and/or BHET, with the most commonly used being reversed‐phase high‐performance liquid chromatography (RP‐HPLC) analysis. One of the earliest fluorometric assays for the detection of PET degradation reported was based on iron autoxidation of TPA to fluorescent 2‐hydroxyterephthalate (Wei et al., 2012). However, other approaches relying on fluorescence (Chaves et al., 2022) or simple absorbance measurements (Arnling Bååth et al., 2020) of PET breakdown products have also been reported. While these methods are all very sophisticated, none of them allows a direct and highly specific assessment of PET degradation in the environment. Therefore, developing smart and easy‐to‐apply biosensors is a main challenge. This would allow identifying the degradation products directly in nature and could lead to the responsible microorganisms. Previous research has identified regulatory circuits and genes involved in the uptake of few of the polymer degradation products. Such genes and especially their promoters can be harnessed to generate very specific biosensors. For instance, several transporters for TPA uptake had been reported (Hosaka et al., 2013; Pardo et al., 2020) together with pathways for its degradation (Kasai et al., 2010; Figure 4). Recently, a study implanted this principle and developed a biosensor for TPA using the transcription factor XylS from *Pseudomonas putida* (Li et al., 2022). By using several rounds of mutagenesis, this transcription factor was optimized to recognize TPA at 10 μM concentration. While this manuscript was under review, yet another study showed that the conversion of TPA to an aldehyde leads to bioluminescence suitable for online monitoring and detection of TPA in living cells (Bayer et al., 2022).

Within this framework, the use of RNAseq‐approaches of strains that degrade oligo‐ and monomers of such polymers will soon result in the identification of additional promoters, which will help to develop very useful biosensors.

#### Synthetic approaches to obtain novel or improved enzymes

Synthetic approaches combining directed evolution, rational design and machine learning‐based engineering are a very innovative strategy to obtain biocatalysts with novel functions (Miller et al., 2022). One example is an engineered protein with two active sites that is able to perform a non‐natural conversion (Alonso et al., 2020). This technology could potentially be used to produce completely novel enzymes acting on the polymers for which we do not have enzymes. However, no such study has been reported in the field of plastic‐degrading enzymes. It is more likely to perform random mutagenesis with current enzymes and evolve them to become multifunctional catalysts.

In the past decade, a number of variants have been reported mainly using already well‐studied enzymes. A very recent review by Wei *e*t al. (2022) summarizes these efforts. For LCC, PET2, TfH as well as for the *Is*PETase, such improved variants have been reported with often significantly increased activities over the parental enzymes (Cui et al., 2021; Nakamura et al., 2021; Tournier et al., 2020; Wei et al., 2016). Among these, *Is*PETaseTM appears to be one of the most active *Is*PETase variants (Brott et al., 2022). Only very recently, yet another success story was published using artificial intelligence (AI) approaches to generate a highly active enzyme designated FAST‐PETase (functional, active, stable and tolerant PETase) (Lu et al., 2022). Nevertheless, none of the studies using the *Is*PETase as a platform has achieved the overall activities of the LCC wildtype enzymes or its variants, *Hi*C or *Tf*Cut_2 (Wei et al., 2022).

## INDUSTRIAL BIOPROCESSES ALREADY ESTABLISHED TO RE‐ AND UPCYCLE PLASTICS

The biological re‐ and upcycling of plastic has been discussed since the first enzymes acting on PET became available. Within this setting, enzymes like LCC and *Is*PETase have been optimized for maximum performance under biotechnological conditions. Probably, the most active enzymes known are the LCC variants LCC^ICCG/WCCG^ (Tournier et al., 2020) and the FAST‐PETase (Lu et al., 2022) which seem to be ideal for industrial PET re‐ and upcycling. Carbios, a French company, has picked up this concept. The Carbios process depends on pretreated PET‐flakes, in which the surface has been increased and the level of crystallinity has been lowered significantly. The employed enzyme converts the low‐crystalline PET into its monomers (TPA and EG) at a temperature of about 72°C and within 24 hours [(Tournier et al., 2020); www. carbios.com]. While this process is still running at the pilot scale, it is clear that it bears a vast potential at several levels. Most importantly, it would allow an upcycling of the monomers into products of higher value, but also back into the polymers. Thus, the lifetime of a single plastic bottle could be increased. The here‐developed process could also be a blueprint for the re‐ and upcycling of other polymers once enzymes become available. While the process in this current developmental stage relies on PET‐bottles, it can be expected that sooner or later, used textile fibres and other forms of PET will end up in the industrial fermenters. The process could offer produce to platform chemicals based on waste that would allow the production of valuable compounds such as vanillin that has recently been demonstrated by a conversion from TPA (Sadler & Wallace, 2021). While vanillin is perhaps not the best‐suited platform chemical, this a very recent example of converting PET into higher value products. A completely different but nevertheless very promising approach had been reported by Tiso *e*t al. (2021). These researchers had converted PET into a medium chain‐length PHA and a novel bio‐based poly(amide urethane) (bio‐PU). Yet another approach would co‐culture a PET‐degrader with a PHB producer to produce bioplastic (Liu et al., 2021b). These are only very recent examples of PET upcycling, and it can be expected that this field will be rapidly developing and other valuable products will be obtained from post‐consumer polymers like PET.

## LARGE AMOUNTS OF ENZYMES WOULD BE REQUIRED TO DEGRADE PET IN THE ENVIRONMENT

Today, large quantities of plastics have been released not only into global oceans, lakes but also into terrestrial sites. Calculations estimate that 60% of all plastics produced were discarded and are accumulating in either landfills or in the environment (Geyer et al., 2017; Lebreton & Andrady, 2019). These landmark studies imply that between 100 and 240 million metric tons of various plastics are released annually into the environment, and this would sum up to 0.5–1 billion tons of plastics within 5 years.

Given that they function as man‐made carbon sink and with respect to the CO_2_ and climate discussion, it should be critically evaluated, if a long‐term storage of large plastic pieces within deep layers of soils or ocean sediments is perhaps the better solution, rather than their incineration. The latter will go in parallel with the release of significant amounts of CO_2_. Nevertheless, and due to the lack of enzymes and microbial pathways acting on most of these polymers (e.g. PP, PS, PA, ether‐based PUR, PVC), the larger plastic fragments (>1 cm) in terrestrial or marine sediments will remain there for indefinite time periods.

In the degradation of PET, ester‐based PUR, PA oligomers, rubber, but also bioplastics, enzymatic processes will most likely be involved in nature over very long time periods. A complete enzymatic degradation is, however, only realistic, if the polymers are ground to microplastics with a much higher accessible surface area, but still lower crystallinity and if secreted enzymes are able to attach to the slightly amorphous polymers for hydrolysis (Figures 3 and 5). Within this framework, we can only speculate on the actual velocity of the reactions under natural conditions. Catalytic turnover rates and enzyme availabilities in nature will be significantly lower compared to those known from the best‐performing wildtype enzymes like the *Ideonella sakaiensis* PET hydrolase (*Is*PETase) and leaf compost cutinase (LCC) or even their improved variants under laboratory conditions. Especially in colder environments, enzyme activities will be at least 10‐ to 1000‐fold lower compared to the optimum temperatures.

We estimated that for the degradation of a pretreated 500 ml PET bottle, 150–500 mg of either pure LCC, *Is*PETase or PET27 enzyme would be needed to degrade it within 24 hours at 30°C assuming that the enzymes remain stable for 24 hours. This estimation is based on activity measurements of recombinant wildtype enzymes in our laboratory and the information that an average PET‐bottle weighs 20 grams equalling 0.103 mol of a PET monomer. Additives are not being considered, as they are not present in most PET‐bottles. The assumption is further solely based on the released amount of TPA at near optimum reaction conditions without considering the available surface area, and that possible product inhibition can occur. This shows that in nature, the needed amounts of enzyme will certainly not become available at the same time and could only be produced over very long time periods. Aiming at a degradation over a time of one year would require 0.4–1.4 mg of enzyme being produced per day, assuming that the enzyme remains stable for 24 hours. Since this is a significant amount of enzyme that would require a very high cell density and high‐level expression in nature, it is unlikely that a bottle can be degraded within years solely by enzymatic breakdown.

While the above‐made calculations only look at a single plastic bottle, they clearly underestimate the problem. The annual production of PET lies at 33 million tons. Based on our previous estimation, it would require 6.5–25 kg of the wildtype enzyme to digest a metric ton of pretreated PET‐waste in 24 hours and 200–850 kg to digest 33 tons. For 33 million tons, it would require 200,000–850,000 tons of enzyme. Consequently, if we assume that between 10% and 60% (equals 3–18 million metric tons) of the annually produced PET is released into nature, it would require at least 20,000 tons of enzyme for the degradation under almost ideal laboratory conditions.

While this calculation is highly hypothetical, it clearly emphasizes the high amount of enzyme, which would be required to degrade and upcycle the annually released amount of PET. Within this framework, it is noteworthy that currently no data are available on the actual global occurrence of known PET‐active enzymes at larger levels and thus, there is no evidence for rapid and fast large‐scale decomposition of PET in nature. Within this framework, a recent study implied that seawater may act with all the metal ions as a chemical catalyst hydrolysing ester bonds in the PET polymers and resolving a plastic bottle within 72 years under tropical conditions (Stanica‐Ezeanu & Matei, 2021). This model however remains to be verified by experimental data.

Consequently, these simple calculations and assumptions imply that enzymatic degradation is not going to solve the global plastics crisis in short time.

## FUTURE BIOTECHNOLOGICAL CHALLENGES

While the environmental plastic pollution certainly is a major challenge, a smart way out of this dilemma is to develop bio‐based strategies for the synthesis of truly biodegradable polymers. Today's bioplastics production equals less than 1% of the overall synthetic polymers produced (European‐Bioplastics, 2022). Thus, increasing their production and use by reducing the use of the fuel‐based polymers at the same time is a main challenge.

Nevertheless, finding enzymes acting at PE, PP, PVC, ether‐based PUR, PA and others is an immediate and urgent task that needs to be addressed.

A further step that should be taken is to increase the performance of the currently known PET‐ and PUR‐active enzymes. Since neither attack high‐density and highly crystalline polymers, it would be ideal to modify them to accept these as substrates and thereby also allow water to enter the dense polymer structure. One step could be performed by developing enzymes that intercalate into the fibre similar to the expansin‐like non‐catalytic proteins used in fungal cellulases (Quarantin et al., 2019).

Besides, the chemical or enzymatic modification of so‐called natural polymers such as chitin, alginate, cellulose, glycogen, PHBs, PHAs, DNA, proteins and/or starch may result in the production of semi‐synthetic polymers that have highly sophisticated biodegradative capabilities. The design of such semi‐synthetic polymers is an attractive target as well, but it does not mean that these polymers are being degraded faster (Narancic et al., 2018).

To further advance in this field, additional biopolymers with improved traits with respect to durability, elasticity and longevity are needed. They must compete in their material and physical properties with those of the synthetic polymers made from fossil fuel. Clearly, increased durability is invariably accompanied by decreasing biodegradability. A possible way out of this dilemma is to modify current polymers by simply introducing breaking points. Otherwise, the implanting of immobilized and stable enzymes into the fibres, which can be activated after some time to initiate the biodegradation, might be one strategy. However, this needs to be done with great care since earlier examples using oxo‐degradable polymers failed (Directorate‐General Environment of the European Commission et al., 2016).

Nevertheless, very promising examples have been published recently for poly‐(L‐lactic acid) (PLA) films and for using embedded proteinase K for self‐degradation (Huang et al., 2020). Recently, a very convincing study has demonstrated that using 3D printing with a lipase implemented in PCL as composite films could be a highly attractive technological advancement (Greene et al., 2021). Furthermore, the implementation of lipase and/or proteinase K as nano‐dispersed enzymes into PCL and PLA films was shown to be a highly efficient and advanced technology for polymer degradation (DelRe et al., 2021). If this can be applied at larger scales and meets the required standards needs to be demonstrated.

At least in theory, it should also be possible to modify the reaction conditions to synthesize a biodegradable PET or ester‐based PUR by reversing the reaction of the known PET‐ or PUR‐active enzymes. Since this reaction would be performed under almost water‐free conditions, enzymes from extremophiles could be the key to the synthesis of such polymers (Antranikian & Streit, 2022). Notably, any biosynthesized PET or PUR will initially not be better degradable than the chemically synthesized ones. Better biodegradability could only be achieved if one of the above‐outlined strategies will be applied.

We will hardly be able to do entirely without plastic materials in the future. With such a multifactorial problem as the global plastic crisis, there is no easy solution. However, the problem is too urgent to be passed on to future generations. It is important that as little plastic waste as possible is created in the first place. To achieve this, companies, lawmakers and the society must develop serious strategies. First steps to be taken in order to operate more sustainably, to reduce the burden on the environment and to become less dependent on fossil raw materials would be, for example, the harsh reduction of single‐use plastics, especially as packaging materials, the replacement of C‐C bond‐based polymers by ester‐based polymers (synthesized primarily from bio‐based materials) as these can be degraded easier (Law & Narayan, 2022) and the avoidance of difficult‐to‐recycle composites. Thereby, the production of plant‐based feedstocks for bioplastics must not compete in the agricultural sector with farmland used for food production.

## AUTHOR CONTRIBUTIONS

W.R.S., J.C., P.P.G. and R. D. designed the study and contributed to manuscript writing. R.D. provided the image for Figure 5B.

## FUNDING INFORMATION

This work was in part supported by the Federal Ministry of Education and Research (BMBF) within the programs LipoBiocat (031B0837B) and PlastiSea (031B867B).

## CONFLICT OF INTEREST

The authors declare no conflict of interest.

## Supporting information


Figure S1
