# Supplementary figures and images for: Microbial enzymes will offer limited solutions to the global plastic pollution crisis
Source: Microb Biotechnol. 2022 Sep 13;16(2):195–217. doi: 10.1111/1751-7915.14135 (PMC9871534; doi:10.1111/1751-7915.14135)

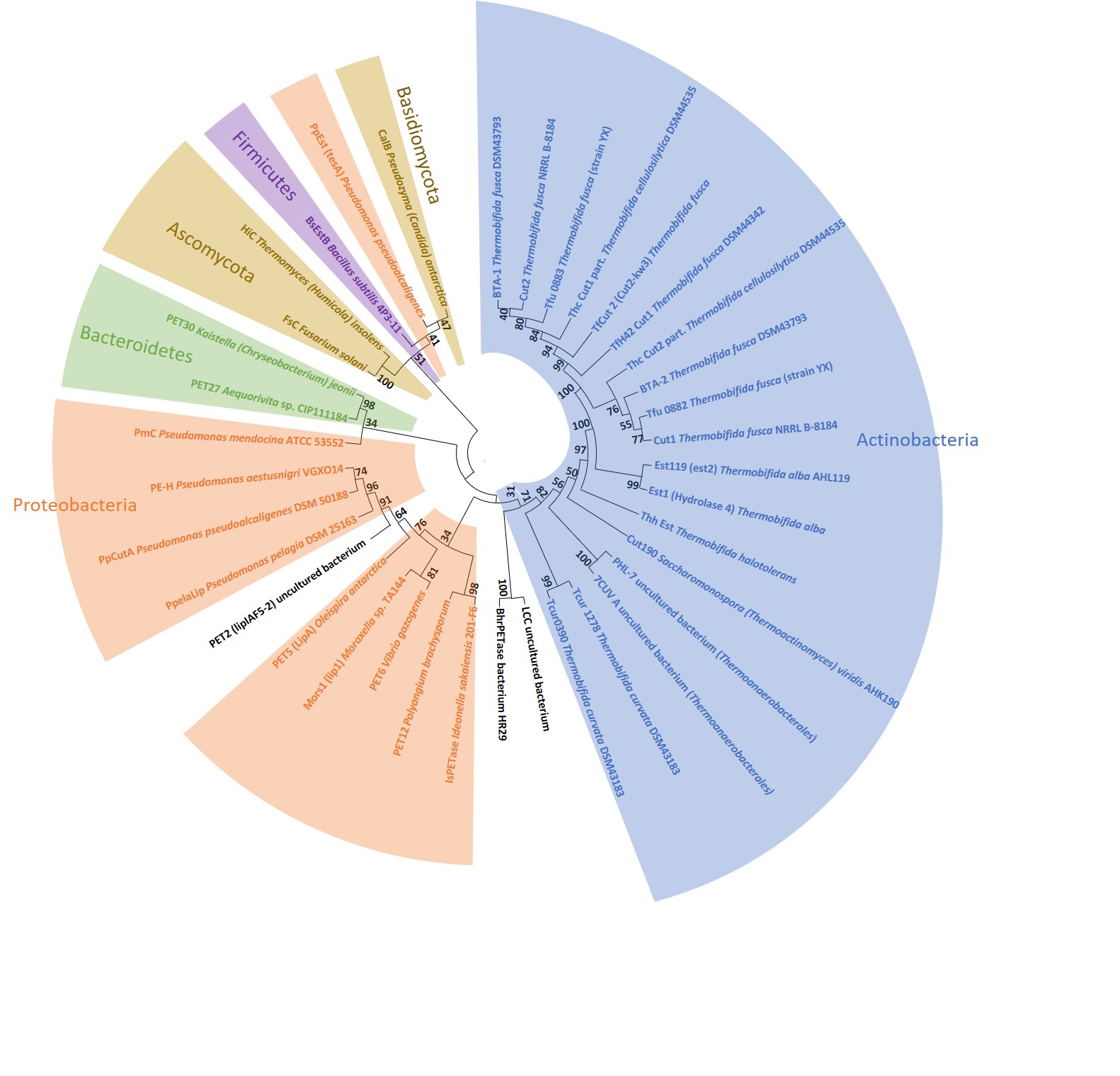

Supplement: Supplementary file 1 — Figure S1 [file MBT2-16-195-s001.jpg]
